# Metaverse‐based virtual reality and spatial learning in cervicofacial anatomy: A curriculum‐embedded comparative study in undergraduate medical students

**DOI:** 10.1002/ase.70277

**Published:** 2026-07-06

**Authors:** Taku Ito, Hidekazu Kawasaki, Ayame Yamazaki, Yoshimaru Mizoguchi, Kumiko Yamaguchi, Eiji Kaneko, Takeshi Tsutsumi

**Affiliations:** ^1^ Department of Otorhinolaryngology Institute of Science Tokyo Bunkyo‐ku Japan; ^2^ School of Medicine Institute of Science Tokyo Bunkyo‐ku Japan; ^3^ Center for Healthcare Education Institute of Science Tokyo Bunkyo‐ku Japan

**Keywords:** anatomy education, cervicofacial anatomy, medical students, metaverse, otorhinolaryngology, spatial learning, virtual reality

## Abstract

To compare a metaverse‐based interactive virtual reality (VR) session with a conventional 2D online lecture for learning cervicofacial anatomy. Single‐center, curriculum‐embedded, nonrandomized comparative study. Fourth‐year students attended a Zoom lecture (Conventional, *n* = 73); sixth‐year students completed an interactive VR session using Meta Quest 2 on the Spatial platform (VR, *n* = 40), delivered in small groups. Immediately after teaching, students completed a 12‐item test (9 spatial‐relational, 3 factual/topographic). Group differences were analyzed with Welch's *t*‐tests. VR students scored higher overall than Conventional students (66.0% ± 18.3% vs. 43.6% ± 18.0%; mean difference 22.4 percentage points, 95% CI 15.3–29.6; *p* < 0.0001), driven by spatial items (68.6% ± 20.1% vs. 40.6% ± 18.9%; difference 28.0, 95% CI 20.3–35.7; *p* < 0.0001). Factual/topographic scores were similar (58.3% ± 25.9% vs. 52.5% ± 30.4%; difference 6.9, 95% CI −3.4 to 17.2; *p* = 0.19). Optional free‐text comments suggested high engagement and perceived spatial benefits; barriers included headset ergonomics and occasional motion sickness. Metaverse‐based interactive VR was associated with substantially better spatial learning than a conventional online lecture, with comparable factual recall. Given the nonrandomized design and small‐group VR delivery, future studies should use size‐matched randomized designs with baseline testing and richer qualitative methods to isolate immersion effects and evaluate generalizability to other anatomical regions.

## INTRODUCTION

Anatomy is a cornerstone of medical education, providing the foundational knowledge necessary for clinical practice. Mastery of anatomical structures requires not only memorization of names and locations, but also a profound understanding of three‐dimensional (3D) spatial relationships between organs, vessels, and nerves. However, conventional lecture‐based approaches, which typically rely on two‐dimensional (2D) images and diagrams, or cadaveric demonstrations that only reveal the periphery in section, often fall short in fostering this spatial comprehension. These limitations are particularly pronounced for novice learners, who may struggle to mentally reconstruct 3D structures from static, flat representations. In this study, we focus on cervicofacial anatomy (ear, paranasal sinuses, and neck) as a spatially complex region that is central to otorhinolaryngology training and well‐suited to interactive 3D learning.

In recent years, advances in virtual reality (VR) and metaverse technologies have created new opportunities for immersive and interactive learning experiences and for surgical planning/clinical visualization.^1^, ^2^, ^3^, ^4^, ^5^ Immersive VR allows learners to explore anatomical structures from multiple perspectives, manipulate models in real time, and engage in bidirectional communication with instructors.^6^ Previous studies on the education of healthcare professionals have suggested that such active learning environments may enhance motivation, engagement, and knowledge retention.^7^, ^8^ Nevertheless, the empirical evidence for the superiority of VR over conventional didactic methods in anatomy education remains limited, and many existing studies have small sample sizes, lack control groups, or do not directly compare immersive VR with traditional formats.^9^

To address these gaps, we developed a metaverse‐based VR anatomy lecture in which both students and instructors entered a shared virtual space as avatars and collaboratively explored detailed 3D anatomical models. We hypothesized that immersive VR would preferentially benefit spatially demanding anatomy tasks, consistent with multimedia learning and constructivist accounts that active manipulation of 3D representations supports spatial cognition.^10^, ^11^ This study aimed to compare the educational effectiveness of this immersive, interactive lecture format with that of a conventional 2D, instructor‐centered lecture. Our primary research question was whether the metaverse‐based VR lecture could improve students' immediate comprehension of anatomical structures and spatial relationships. As a secondary aim, we explored student perceptions of learning motivation, spatial understanding, and usability of the VR environment.

## METHODS

### Study design and ethics

We conducted a single‐center, curriculum‐embedded, nonrandomized comparative study using intact cohorts. Fourth‐year students attended a conventional online lecture (Conventional), and sixth‐year students attended an interactive metaverse‐based VR session (VR). Otorhinolaryngology residents (Resident) completed the same assessment as a benchmark cohort to contextualize performance and to confirm item appropriateness. The study protocol was reviewed and approved by the Institutional Review Board of the Institute of Science Tokyo (Approval No. E2025‐049), and informed consent was obtained as required.

### Participants and setting

Participants were undergraduate medical students enrolled in scheduled, credit‐bearing otorhinolaryngology curriculum sessions and otolaryngology residents at a single institution. Attendance was required as part of the curriculum. Students belonged to two pre‐existing cohorts: fourth‐year students who received the conventional Zoom lecture (Conventional) and sixth‐year students who received the metaverse‐based VR lecture (VR); thus, participants were not randomized. After the session, students were asked to complete an anonymous assessment; participants could decline use of their data for research. All participants had completed standard anatomy coursework. Under the revised curriculum, neither cohort had received a comparable otorhinolaryngology anatomy session covering the cervicofacial structures targeted in this intervention during the same academic year; accordingly, the sixth‐year cohort had not previously taken an equivalent fourth‐year lecture on this content. No participant reported prior VR experience. Because the training stage differed between cohorts, we prespecified analyses focusing on item type (spatial vs. factual) and the within‐participant difference score (Δ = Spatial−Factual, percentage points) to mitigate baseline differences in overall ability.

### Lecture formats and materials

**FIGURE 1:**
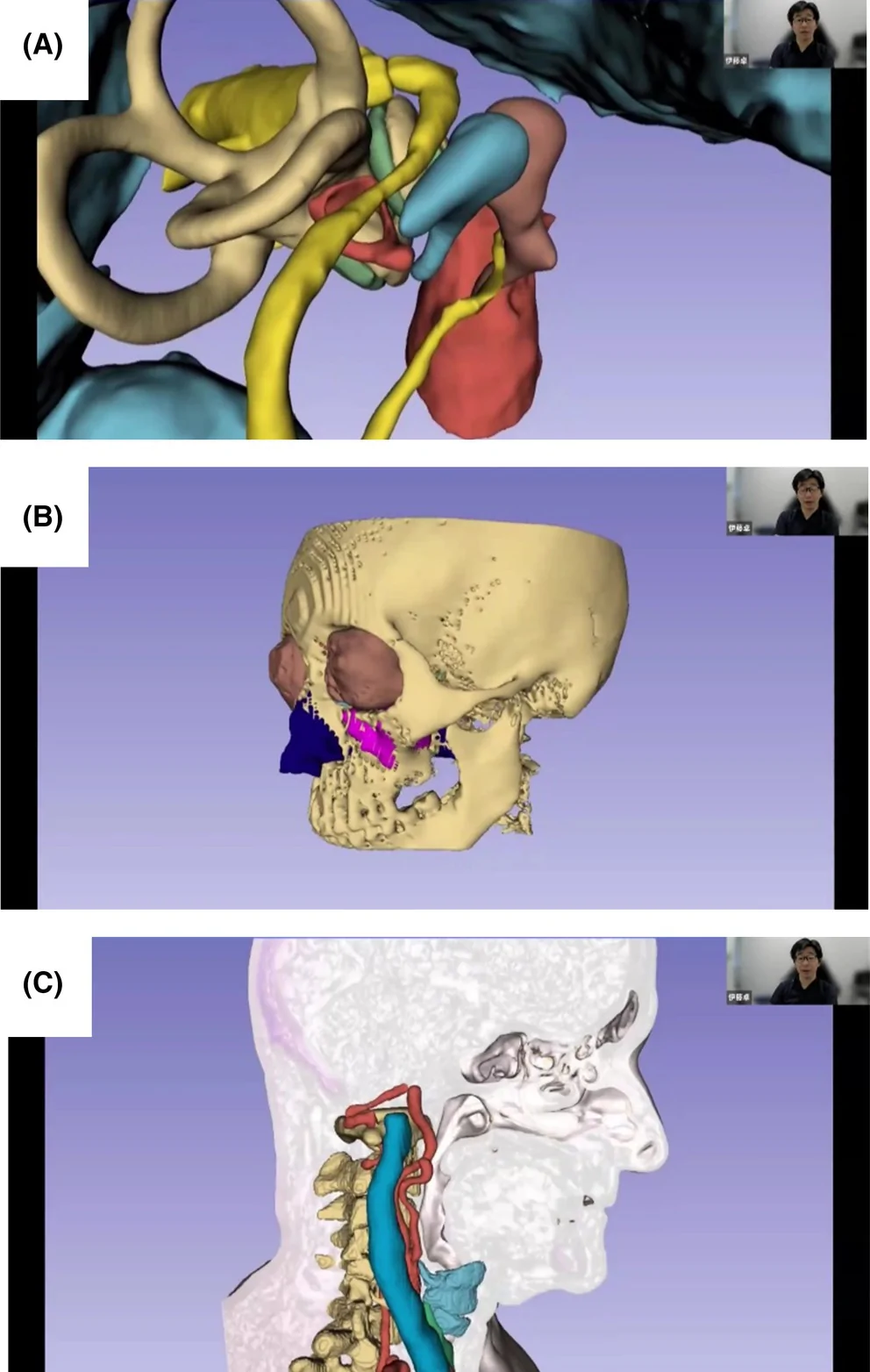
Conventional 2D lecture (Zoom) and representative anatomy views. Still frames from the conventional, instructor‐led 2D lecture delivered via Zoom. The instructor screen‐shared manipulable 3D models while students viewed them on flat displays. Panels illustrate the major anatomical regions covered in the session: (A) temporal bone/otologic anatomy (semicircular canals, cochlea, facial/chorda tympani nerve, adjacent vessels), (B) skull and paranasal sinus anatomy, and (C) cervical anatomy (airway and major vessels).

Conventional lecture: The conventional session was delivered to fourth‐year medical students during the 80‐min course “Otorhinolaryngology Mini Case,” conducted live via Zoom (Figure 1). The anatomy component analyzed in this study consisted of an approximately 20‐min instructor‐led segment (with brief Q&A), during which the instructor screen‐shared the 3D anatomical models; students did not manipulate the models themselves.

**FIGURE 2:**
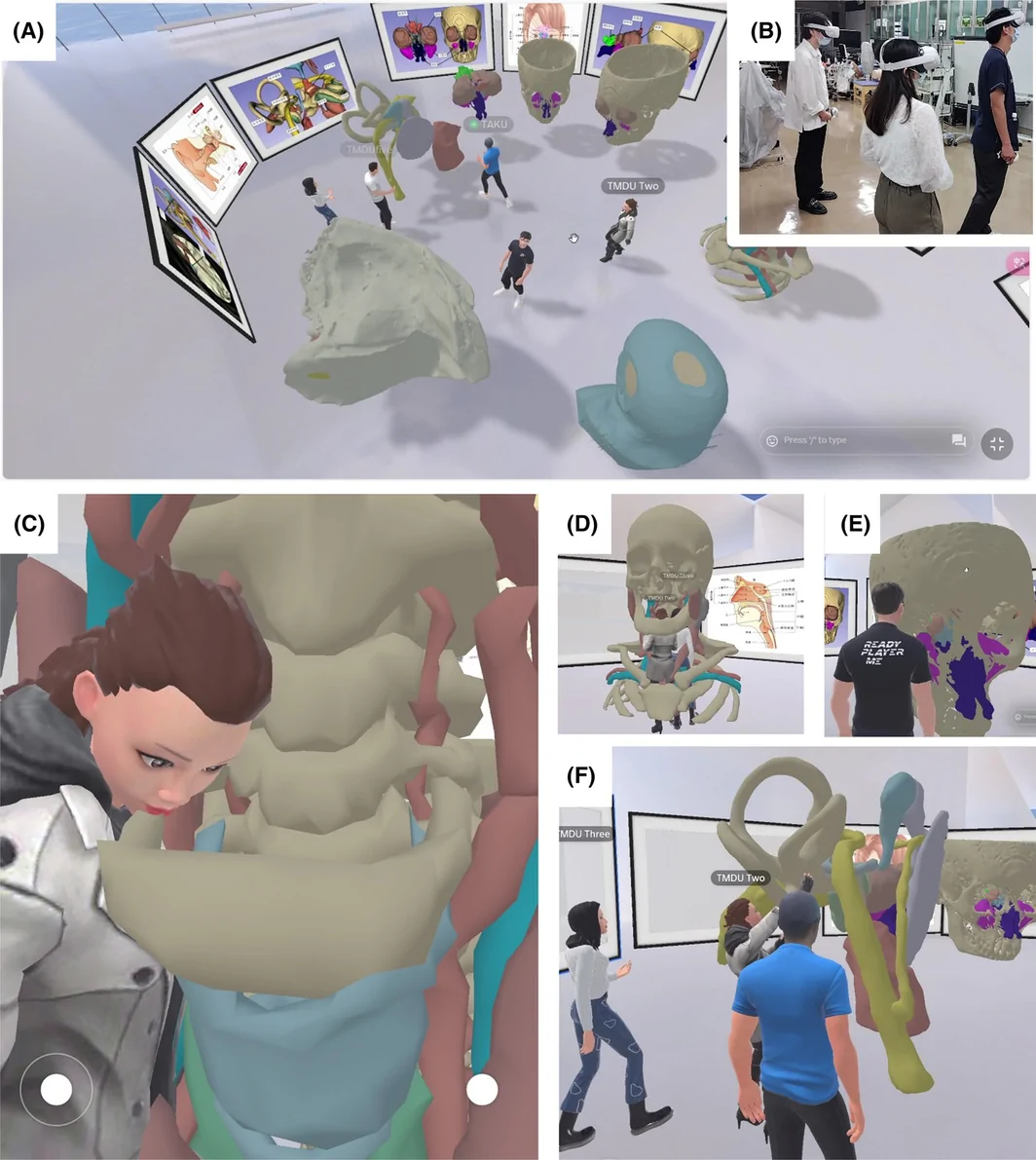
Metaverse‐based VR learning environment and interactions. Scenes from the metaverse VR session (Spatial platform; Meta Quest 2 head‐mounted displays). (A) Shared virtual classroom where students and instructors enter as avatars and gather around large‐scale 3D models. (B) On‐site view of participants wearing headsets. (C–F) Examples of embodied exploration and collaboration: Walking inside models to inspect deep anatomical relationships, rotating/scaling structures, and discussing labeling/orientation in real time.

Metaverse‐based VR lecture: The VR session was delivered to sixth‐year medical students as part of the otorhinolaryngology and head & neck surgery clinical clerkship program “VR Anatomy Practicum.” Using Meta Quest 2 head‐mounted displays on the Spatial platform, the lecture was repeated in small groups of 2–3 learners per session due to device availability (Figure 2). Each session comprised an approximately 20‐min guided exploration followed by a brief open discussion, during which students and the instructor collaboratively manipulated the models in a shared virtual space.

### Learning materials

All anatomical models included relevant organs, vessels, and nerves at a level of detail suitable for medical students. Models could be scaled, rotated, and dissected virtually to reveal internal structures. For example, middle ear models allowed inspection of ossicles, associated nerves, and surrounding bony landmarks from any orientation. The VR content covered the same anatomical topics and learning objectives between the conventional and VR lectures.

### Outcome measures

#### Anatomy comprehension test

Immediately after each lecture, participants completed a 12‐item single‐best‐answer test assessing cervicofacial anatomy. Items were prespecified as Spatial–relational (9/12 items; Q1–5, Q9–12) or Factual/topographic (3/12 items; Q6–8) based on whether they required mental manipulation of 3D anatomy (e.g., depth/orientation/adjacency) versus recall of discrete anatomical information (see Supplemental Methods). Outcomes were summarized as percent‐correct overall and by item type. The primary student‐group analysis was the within‐participant difference score Δ = (percent correct on Spatial–relational items) − (percent correct on Factual/topographic items), compared between the VR and Conventional cohorts using Welch's *t*‐test with Hedges' *g*. Items covered three regions: ear (5 items), nose/paranasal sinuses (3 items), and neck (4 items). Each correct answer scored 1 point (maximum 12).

#### Student questionnaire (VR cohort only)

Following the VR session, students were invited to complete an anonymous post‐session perception questionnaire (voluntary). Items 1–3 assessed (i) self‐reported understanding of the cervicofacial anatomy content; (ii) evaluation of the metaverse‐based session format; and (iii) willingness to participate again, using 5‐point Likert‐type response options. Items 4–6 were open‐ended and asked about perceived advantages, disadvantages, and suggestions for improvement. Verbatim items and response anchors are provided in Data S2. Likert‐type response distributions are shown in Figure 5. Structured‐response options and the thematic summary of optional free‐text comments are provided in Table S8. Free‐text comments were optional. Open‐ended responses were summarized using an inductive content/thematic approach and are reported descriptively, given the limited response rate; representative excerpts were selected to illustrate each theme.

#### Statistical analysis

The prespecified primary comparison was the between‐cohort difference (VR vs. Conventional) in the within‐student spatial minus factual score difference (Δ, percentage points). Δ was compared using Welch's *t*‐test and summarized with mean differences (95% CI) and Hedges' *g*. As secondary outcomes, we compared Total, Spatial, and Factual percent‐correct scores between cohorts using Welch's *t*‐tests. Comparison of correct response proportions for individual items was performed using Fisher's exact tests (Holm‐adjusted for multiple comparisons). Item‐level results and additional psychometric indices (KR‐20, item difficulty, corrected item–total correlations) and sensitivity analyses (including item‐level models) are provided in the Supplemental Methods and Results. Statistical analyses were conducted in Python 3.11.9 using NumPy, SciPy, pandas, and statsmodels. All tests were two‐sided with *α* = 0.05.

## RESULTS

### Participant characteristics

Participant numbers by cohort are shown in Tables S1 and S2 (Conventional *n* = 73; VR *n* = 40; Residents *n* = 10). Because cohorts were curriculum‐determined and not randomized, we did not attempt detailed demographic matching; related biases are addressed in the Limitations.

### Anatomy comprehension test

**FIGURE 3:**
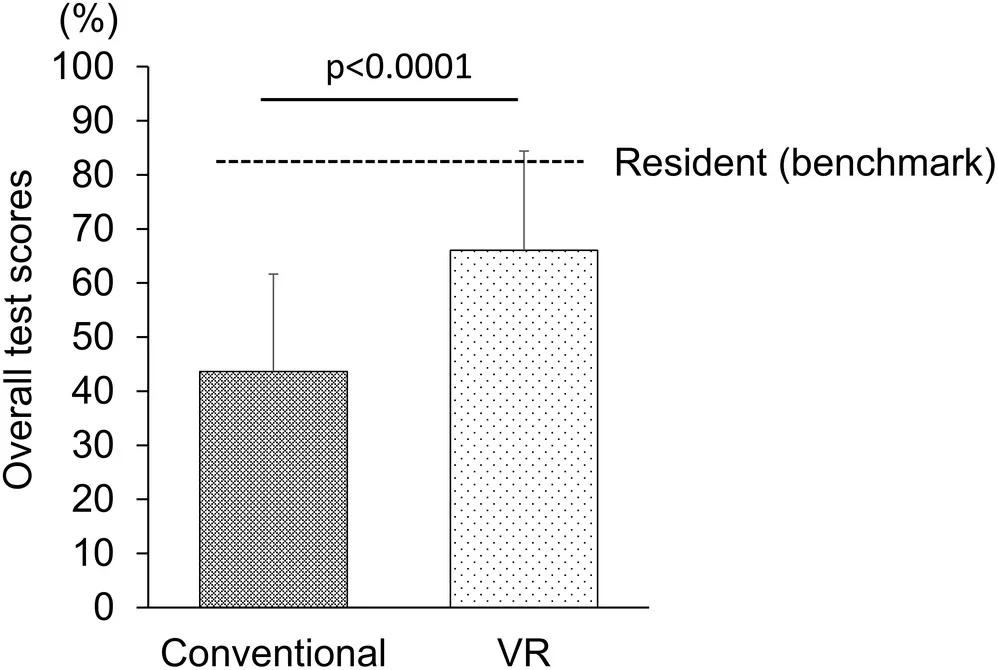
Post‐instruction anatomy test performance by cohort. Mean (±SD) percent correct on the 12‐item anatomy test for students taught by the conventional 2D lecture (Conventional; *n* = 73) and the metaverse‐based VR session (VR; *n* = 40). The dotted horizontal line indicates the resident benchmark (Resident; *n* = 10). Error bars indicate SD.

VR students scored higher than Conventional students on total percent‐correct (mean ± SD: 66.0 ± 18.3 vs. 43.6 ± 18.0; mean difference 22.4 [95% CI 15.3–29.6], Welch's *t*‐test *p* < 0.0001; Hedges' *g* = 1.23) (Figure 3). Resident results are summarized descriptively in Table S2.

**FIGURE 4:**
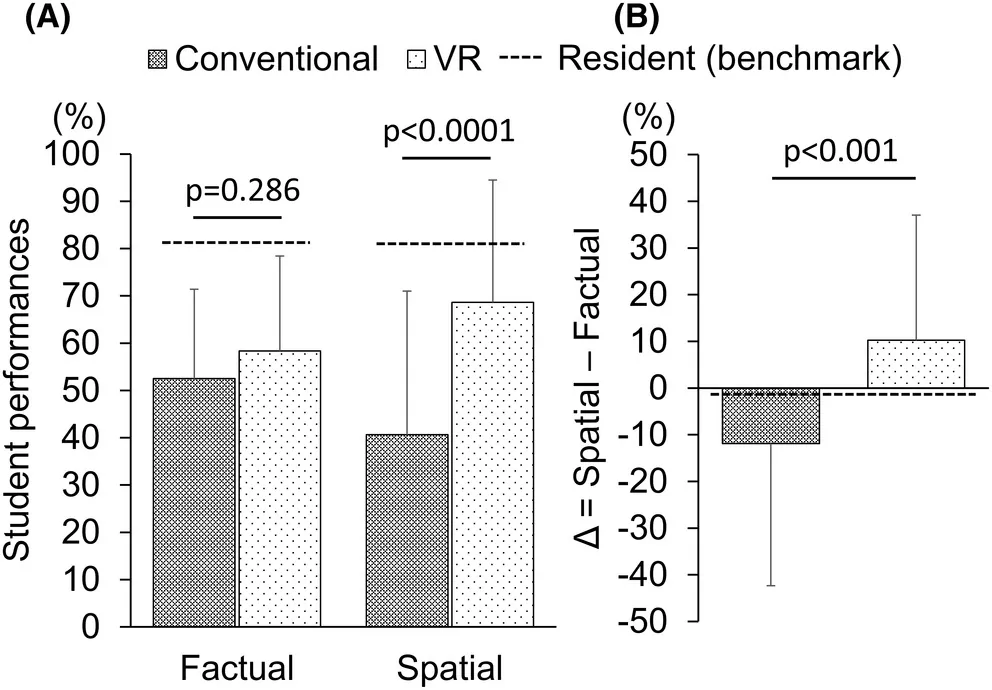
Student performance by item type and within‐student spatial–factual contrast. (A) Mean (±SD) per‐student percent correct for spatial–relational items (9 items: Q1–5, Q9–12) and factual/topographic items (3 items: Q6–8) in the Conventional (*n* = 73) and VR (*n* = 40) cohorts. The dotted horizontal line indicates the resident benchmark (Resident; *n* = 10), shown for reference. (B) Within‐student difference score, Δ = (Spatial–relational percent correct) − (Factual/topographic percent correct), shown as mean (±SD). Values of Δ > 0 indicate better performance on spatial–relational items relative to factual/topographic items. Error bars indicate SD.

By item type, the VR advantage was pronounced for spatial items (mean ± SD: 68.6 ± 20.1 vs. 40.6 ± 18.9; mean difference 28.0 [95% CI 20.3–35.7], Welch's *t*‐test *p* < 0.0001; Hedges' *g* = 1.44), whereas factual/topographic items were similar between groups (58.3 ± 25.9 vs. 52.5 ± 30.4; difference 5.8 [−4.9–16.6], *p* = 0.286; *g* = 0.20) (Figure 4A). Consistent with this pattern, the within‐student spatial–factual difference (Δ = Spatial−Factual) shifted from negative in the Conventional cohort to positive in the VR cohort (mean Δ: −11.9 ± 30.4 vs. +10.3 ± 26.8; between‐group difference 22.1 [11.2–33.1], *p* < 0.001), indicating a selective benefit of VR for spatial reasoning (Figure 4B). Item‐level results and sensitivity analyses are provided in Table S3. Item‐level GEE analyses corroborated a significant cohort × item‐type interaction favoring VR on spatial items; additional model details and psychometric indices are reported in the Supplemental Methods and Tables S4–S7.

### Student questionnaire and qualitative feedback (VR cohort only)

**FIGURE 5:**
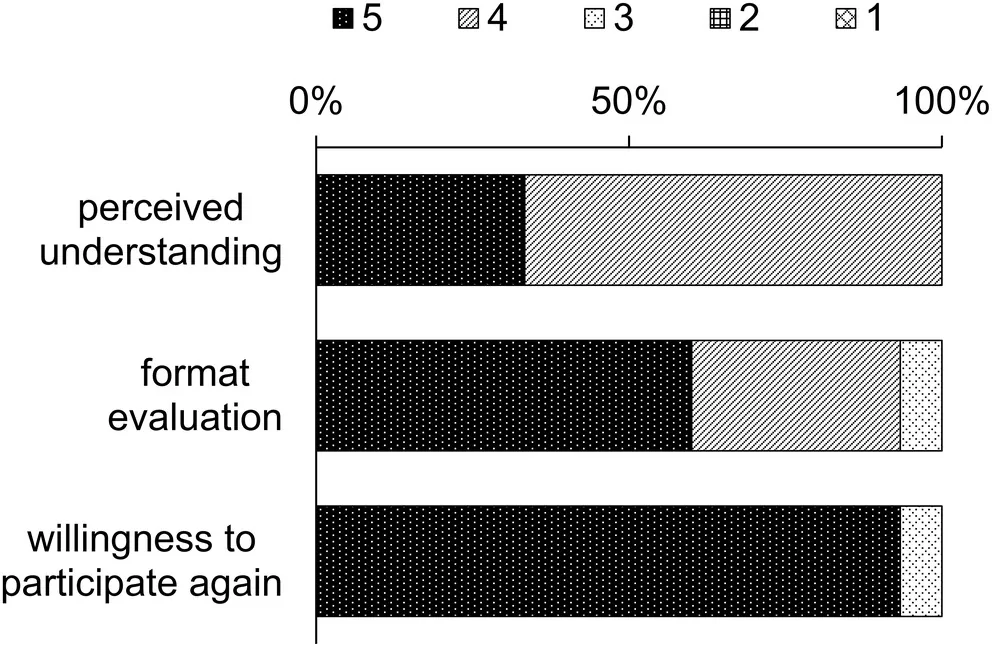
VR student questionnaire (5‐point Likert) responses. Stacked horizontal bars show the distribution of responses among VR participants (*n* = 15). Response options differed by item: Perceived understanding (1 = Not understood at all to 5 = Very well understood), metaverse class format evaluation (1 = Very negative to 5 = Very positive), and willingness to participate again (1 = Strongly no to 5 = Strongly yes). Verbatim items are provided in Data S2.

**FIGURE 6:**
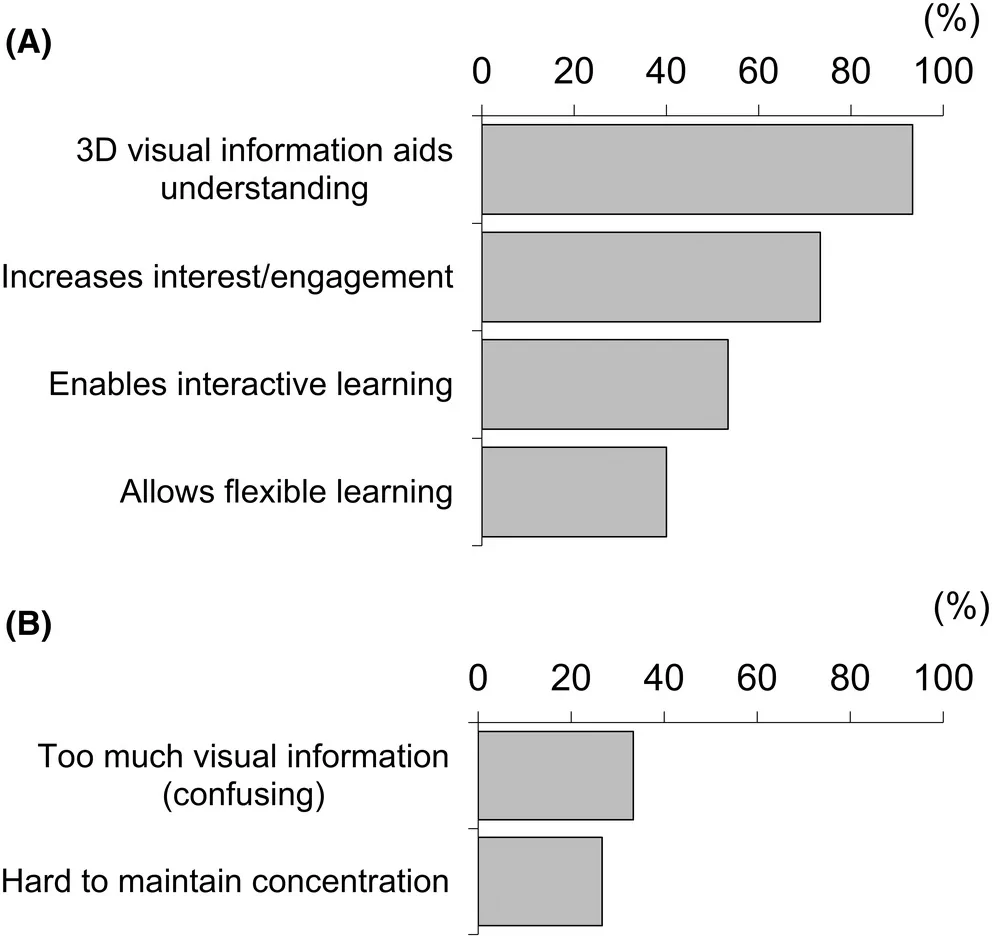
Structured feedback from the VR cohort. (A) Perceived advantages of the metaverse‐based class and (B) perceived disadvantages (multiple selections allowed for each item). Bars represent the percentage of respondents (denominator: 15 students who completed the questionnaire) selecting each option; totals may exceed 100% because multiple responses were permitted. Additional low‐frequency disadvantage options are reported in Table S8.

Of the 40 students who participated in the VR session, 15 voluntarily completed the post‐session questionnaire. Overall, respondents reported high self‐reported understanding of the cervicofacial anatomy content, positive evaluations of the metaverse‐based session format, and willingness to participate again (Figure 5; *n* = 15; see Data S2 for verbatim items/anchors and Table S8 for qualitative themes and low‐frequency responses). Open‐ended responses regarding perceived advantages and disadvantages were categorized and summarized descriptively (Figure 6), and less frequently mentioned responses were tabulated in Table S8. Optional free‐text comments (Item 6) were summarized thematically with representative excerpts (Table S8) and visualized as a word cloud (Figure S1).

## DISCUSSION

These findings suggest that immersive, metaverse‐based VR instruction is associated with higher performance on spatial–relational anatomy items, whereas factual/topographic scores were comparable between cohorts. VR and AR have been increasingly adopted in medical and health sciences education, with evidence suggesting benefits particularly for learning tasks that depend on spatial visualization and interaction with 3D representations.^2^, ^3^, ^6^, ^12^, ^13^ In anatomy education specifically, recent systematic reviews and meta‐analyses have converged on small‐to‐moderate learning gains with immersive technologies, while emphasizing heterogeneity in intervention design, outcome measures, and study quality.^6^, ^9^, ^12^, ^13^

To partially address the training‐stage imbalance (fourth‐ vs. sixth‐year), we prespecified a within‐student contrast (Δ = Spatial − Factual) that targets a modality × item‐type pattern rather than overall ability. Nonetheless, the quasi‐experimental, nonrandomized design precludes causal inference, and residual confounding cannot be excluded because grade level and cohort were perfectly confounded. To further probe cohort effects, we conducted an item‐level GEE controlling for anatomical region, which demonstrated a significant cohort × item‐type interaction favoring VR. Together with the between‐cohort difference in Δ, these convergent analyses support the interpretation that the observed advantage is selective to spatial–relational learning rather than a uniform improvement across item types. Residents showed near‐ceiling performance and no systematic spatial–factual imbalance, which is consistent with the notion that the two‐item categories were not trivially separated by difficulty alone.

Our results are consistent with prior studies showing that immersive 3D environments can improve learners' ability to apprehend complex spatial relationships in anatomy.^6^, ^7^, ^12^, ^13^ A randomized controlled study of a 3D ear model previously demonstrated measurable improvements in otologic anatomy learning, supporting the plausibility of spatially oriented benefits.^7^ In addition, visuospatial factors—such as stereopsis and baseline spatial ability—are known to influence who benefits most from 3D/VR learning, which may partially explain variability across studies and cohorts.^14^, ^15^, ^16^, ^17^ Future trials should therefore consider measuring spatial ability and stratifying or adjusting analyses accordingly.^14^, ^15^, ^16^

A key distinction of our intervention is the collaborative “metaverse” format: learners and instructors occupied the same virtual space as avatars and co‐manipulated shared models in real time. Collaborative, interactive VR may support not only spatial comprehension but also broader engagement and “twenty‐first century skills” (e.g., communication and collaboration), which have been highlighted in general‐education VR/AR research.^1^ Such bidirectional interaction may also influence motivation to learn, a construct grounded in contemporary motivational theory and known to shape learning behaviors and outcomes.^8^ In our cohort, optional narrative feedback aligned with this interpretation, with students describing high engagement and perceived benefits for understanding spatial relationships. Similar collaborative affordances have been reported in mixed‐reality simulation and distance‐learning contexts, suggesting that interactivity and social presence may be important active ingredients beyond immersion alone.^14^

From an educational theory perspective, these findings are compatible with constructivist accounts of learning through active exploration and social interaction, as well as multimedia‐learning principles that emphasize selecting, organizing, and integrating information across channels.^10^, ^11^ They are also consistent with embodied/spatial‐cognition frameworks proposing that physical experience (or its functional equivalent via embodied interaction) can enhance understanding of spatially structured concepts.^18^ Notably, this theoretical framing also explains why improvements may be more pronounced for spatial–relational items than for discrete factual/topographic recall, which can be less dependent on interactive 3D manipulation.^11^, ^16^

Despite these benefits, implementation challenges were evident. Students reported barriers, including headset ergonomics and occasional motion sickness. Cybersickness is a well‐described limitation in VR applications and can constrain usability and session duration.^19^ In addition, the richness of immersive visual input can increase cognitive load, highlighting the need to manage working‐memory demands through pacing, segmentation, and scaffolding.^20^ Practical mitigation strategies include scheduled breaks, explicit orientation cues (anterior–posterior/superior–inferior/medial–lateral), and ergonomic hardware optimization.^19^, ^20^

This work also builds on our prior experience using patient‐specific VR/MR for temporal bone anatomy education and surgical planning, and on ongoing efforts to integrate AR into otologic and skull base surgery workflows.^4^, ^5^ Together, these lines of work support a broader view that immersive and interactive 3D technologies can play complementary roles across the continuum from foundational anatomy learning to procedural planning and intraoperative support.^4^, ^5^

## LIMITATIONS

This study has several limitations. First, the study used intact, nonrandomized cohorts at different stages of training (fourth‐ vs. sixth‐year), and training stage was therefore perfectly confounded with group assignment; although our within‐student Δ contrast (Spatial − Factual) targets a modality × item‐type pattern rather than overall ability, residual confounding cannot be excluded. Second, the instructional conditions differed not only in medium (2D online vs. immersive VR) but also in delivery format and interaction opportunity: the conventional condition was delivered to a single large cohort (*n* = 73) in one synchronous session, whereas the VR condition was delivered repeatedly in small groups (2–3 learners per session), with substantially greater opportunity for direct instructor interaction and discussion. Therefore, the observed differences may partly reflect instructor–learner ratio and interactivity rather than immersion alone. An ideal comparative design would deliver the control condition in similarly sized small Zoom‐based groups to equalize interaction time and opportunity across formats. Third, we assessed only immediate post‐session comprehension; delayed retention and transfer were not evaluated. Fourth, test reliability for students was moderate (KR‐20 = 0.61) with a 12‐item pool, constraining measurement precision; resident reliability estimates were unstable owing to a small sample and near‐ceiling performance. Finally, this single‐center study focused on cervicofacial anatomy and may not generalize to other contexts without further validation.

## CONCLUSION

This study provides evidence that a metaverse‐based virtual reality (VR) anatomy lecture can significantly enhance medical students' spatial understanding and learning motivation compared with a conventional didactic format. By enabling immersive, interactive exploration of three‐dimensional anatomical structures, the VR approach addressed limitations inherent in 2D lecture methods and offered unique opportunities for collaborative learning. While challenges related to information overload, headset ergonomics, and motion sickness remain, the potential of metaverse‐based VR to complement and enrich anatomy education is clear. Further research should focus on optimizing instructional design, evaluating long‐term retention, and expanding implementation to diverse medical educational contexts.

## AUTHOR CONTRIBUTIONS

**Kumiko Yamaguchi:** Resources. **Yoshimaru Mizoguchi:** Methodology. **Eiji Kaneko:** Resources; writing – review and editing. **Hidekazu Kawasaki:** Methodology; investigation. **Takeshi Tsutsumi:** Resources; writing – review and editing; supervision. **Ayame Yamazaki:** Methodology; writing – review and editing. **Taku Ito:** Conceptualization; methodology; formal analysis; investigation; resources; writing – original draft; data curation; project administration; validation; visualization.

## FUNDING INFORMATION

This research received no specific grant from any funding agency in the public, commercial, or not‐for‐profit sectors.

## CONFLICT OF INTEREST STATEMENT

The authors declare no competing interests.

## Supporting information


**Supporting Information S1.** Supplemental Methods

**Data S1.** Anatomy comprehension test items

**Data S2.** Student perception questionnaire items

**Table S1.** Cohort characteristics and instructional conditions. Summary of participant cohorts and teaching formats. The VR session was delivered repeatedly in small groups of 2–3 learners per session due to headset availability; therefore, interaction time and discussion intensity may differ from the conventional whole‐cohort lecture. Residents participated in the assessment only as a benchmark cohort.
**Table S2.** Descriptive performance by item type across cohorts. Mean ± SD percent correct for spatial‐relational and factual/topographic item categories for the Conventional (*n* = 73), VR (*n* = 40), and Resident (*n* = 10) cohorts. Values are descriptive summaries corresponding to Figures 3 and 4.
**Table S3.** Item‐level accuracy for students (VR vs. Conventional). Item‐level correctness is shown as number correct/total (%) for each cohort. Between‐cohort differences were tested using two‐sided Fisher's exact tests, with Holm‐adjusted p‐values across 12 items. Odds ratios (ORs) >1 favor VR; 95% CIs were computed with a Haldane–Anscombe correction when any cell count was zero.
**Table S4.** Internal consistency (KR‐20) of the 12‐item test. KR‐20 is reported for pooled students and by cohort, with 95% bootstrap confidence intervals (2000 iterations). Resident reliability is estimated with high uncertainty due to a small sample size (*n* = 10) and near‐ceiling performance, which can attenuate variance and yield unstable KR‐20 estimates.
**Table S5.** Item‐level GEE for student accuracy (VR vs Conventional). Population‐averaged GEE (binomial logit) of item‐level correctness among students (VR and Conventional). Predictors included cohort (VR vs. Conventional), item type (Spatial vs. Factual), their interaction, and anatomical region fixed effects (Ear as reference). Student was the clustering unit with an exchangeable working correlation and robust (sandwich) standard errors. Results are reported as coefficients (*β*), ORs, 95% CIs, and p‐values.
**Table S6.** GEE model specification and estimated within‐student correlation (*ρ*). Model specification for the student item‐level GEE (binomial/logit), including outcome, predictors, clustering unit, working correlation structure, estimated within‐student correlation (*ρ*), and sample sizes (observations and clusters).
**Table S7.** Robustness checks for the student GEE across working‐correlation structures. Sensitivity analyses using alternative working‐correlation structures (exchangeable, independence, and AR (1)). The VR × item‐type interaction is summarized as OR (95% CI), *z*, and *p* for each structure; the corresponding within‐student correlation estimate (*ρ*) is reported where applicable. Collinearity is summarized as the maximum variance inflation factor (VIF).
**Table S8.** Thematic summary of optional free‐text comments from VR participants. Themes, subthemes, and representative comments (English translation/summary) derived from the optional free‐text field of the VR questionnaire. Comments were analyzed using an inductive thematic/content analysis approach and are presented descriptively.
**Figure S1.** Word cloud of optional free‐text comments (Q6) from the VR cohort. The word cloud visualizes recurring concepts expressed in the optional free‐text field. Text was processed by removing common stop words and consolidating obvious synonyms (e.g., “VR”/“metaverse”); the relative word size reflects frequency of appearance across comments. This visualization is intended as a descriptive complement to the thematic summary and representative excerpts and should not be interpreted as a standalone measure of importance.

## Data Availability

The assessment instrument (English translation of the 12‐item anatomy comprehension test) is provided as Data S1 (without the answer key to protect assessment integrity). De‐identified item‐level accuracy data and de‐identified questionnaire response data (quantitative items; free‐text responses available in redacted form if requested) are available from the corresponding author upon reasonable request and subject to institutional data‐sharing policies. No other proprietary datasets were used.
