# Supplementary material for: Metaverse‐based virtual reality and spatial learning in cervicofacial anatomy: A curriculum‐embedded comparative study in undergraduate medical students
Source: Anat Sci Educ. 2026 Jul 6;19(9):1452–9. doi: 10.1002/ase.70277 (PMC13545329; doi:10.1002/ase.70277)
Supplement: Supplementary file 1 [file ASE-19-1452-s002.docx]

**Supplemental Materials**

Title of the article: Metaverse-Based Virtual Reality and Spatial Learning in Cervicofacial Anatomy: A Curriculum-Embedded Comparative Study in Undergraduate Medical Students

A short running title: Metaverse-Based VR for Cervicofacial Anatomy

Authors’ full names: Taku Ito MD, PhD^1^, Hidekazu Kawasaki^2^, Ayame Yamazaki MD, PhD ^1^, Yoshimaru Mizoguchi MD^1^, Kumiko Yamaguchi MD, PhD^3^, Eiji Kaneko MD, PhD^3^, Takeshi Tsutsumi MD, PhD ^1^

Author affiliations:

^1^ Department of Otorhinolaryngology, Institute of Science Tokyo, Tokyo 113 - 8510, Japan

^2^ School of Medicine, Institute of Science Tokyo, Tokyo 113 - 8510, Japan

^3^ Center for Healthcare Education, Institute of Science Tokyo, Tokyo 113 - 8510, Japan

Correspondence: Taku Ito, MD, PhD

Department of Otorhinolaryngology, Institute of Science Tokyo, 1-5-45 Yushima, Bunkyo-ku Tokyo, 113-8510 Japan

Tel: +81-3-5803-5308; E-mail: [taku.oto@tmd.ac.jp](mailto:taku.oto@tmd.ac.jp)

Supplemental Methods

*Item-classification rubric (Spatial-relational vs Factual/topographic).*

Prior to scoring and analysis, we prespecified an item-classification rubric to separate questions that primarily assess spatial reasoning from those that primarily assess factual/topographic knowledge. Spatial-relational items were defined as questions requiring mental manipulation of 3D anatomy (e.g., judging anterior-posterior depth, superior-inferior orientation, relative adjacency, or viewpoint-dependent relationships). Factual/topographic items were defined as questions primarily requiring recall or identification of discrete anatomical information without substantial mental rotation (e.g., nomenclature, ordered lists, or topographic facts). Using this rubric, nine items were classified as Spatial-relational (Q1-5, Q9-12) and three items as Factual/topographic (Q6-8). The rubric and item list are provided in Supplemental Data 1. This a priori separation was intended to enable a transparent item-type analysis of whether the VR format preferentially benefits spatial understanding. The rubric was informed by multimedia-learning and constructivist perspectives and by prior anatomy-education work that distinguishes spatial from non-spatial knowledge in assessment (1-7).

Item-type classifications were reviewed independently by two investigators and finalized by consensus before the dataset was scored; disagreements were resolved through discussion with reference to the learning objectives and required cognitive operations.

*Psychometric evaluation (reliability and validity).*

Items were content-validated by two board-certified otolaryngologists prior to administration. Internal consistency of the dichotomously scored 12-item test was evaluated using KR-20 (equivalent to Cronbach’s alpha for dichotomous items). We report KR-20 estimates for pooled student cohorts and descriptively for residents. Item diagnostics included item difficulty (p) and corrected item-total correlations to assess discrimination. Detailed results are provided in Supplemental Table S4.

*Sensitivity analyses (item-level models).*

To probe potential cohort confounding and to evaluate whether the VR effect differed by item type, we conducted an item-level generalized estimating equation (GEE) with a binomial logit link, with student as the clustering unit and an exchangeable working correlation. Predictors included cohort (VR vs Conventional), item type (Spatial vs Factual), their interaction (primary estimand), and anatomical region fixed effects (Ear as reference). Robust (sandwich) standard errors were used. Because the “Nose” region perfectly overlapped with Factual items, it was omitted to avoid collinearity; grade level was perfectly confounded with cohort and therefore not identifiable in the same model. Model specification, coefficients, and robustness checks are provided in Supplemental Tables S5–S7. Results from alternative working-correlation structures are summarized in Supplemental Tables S5–S7. The estimated within-student correlation (ρ) is reported in Supplemental Table S6.

Supplemental References:

1.	Bruner JS. Toward a theory of instruction: Harvard University Press; 1966.

2.	Mayer RE. Multimedia Learning. 2nd ed. Cambridge: Cambridge University Press; 2009.

3.	Schubert S, Schnabel KP, Winkelmann A. Assessment of spatial anatomical knowledge with a ‘three-dimensional multiple choice test’ (3D-MC). Medical teacher. 2009;31(1):e13-e7.

4.	Yammine K, Violato C. A meta-analysis of the educational effectiveness of three-dimensional visualization technologies in teaching anatomy. Anatomical sciences education. 2015;8(6):525-38.

5.	Langlois J, Bellemare C, Toulouse J, Wells GA. Spatial abilities and anatomy knowledge assessment: A systematic review. Anatomical sciences education. 2017;10(3):235-41.

6.	Roach VA, Mi M, Mussell J, Van Nuland SE, Lufler RS, DeVeau KM, et al. Correlating Spatial Ability With Anatomy Assessment Performance: A Meta-Analysis. Anatomical sciences education. 2021;14(3):317-29.

7.	Dickson J, Shaw DJ, Gardiner A, Rhind S. Testing anatomy: Dissecting spatial and non-spatial knowledge in multiple-choice question assessment. Anatomical sciences education. 2024;17(1):102-13.
