# Supplementary material for: Metaverse‐based virtual reality and spatial learning in cervicofacial anatomy: A curriculum‐embedded comparative study in undergraduate medical students
Source: Anat Sci Educ. 2026 Jul 6;19(9):1452–9. doi: 10.1002/ase.70277 (PMC13545329; doi:10.1002/ase.70277)
Supplement: Supplementary file 2 [file ASE-19-1452-s003.docx]

**Supplemental Data 2. Student Perception Questionnaire Items**

Instructions:
Items 1–3 were rated on a 5-point Likert scale (select one response). Items 4–6 were open-ended.

1. *To what extent did you understand the anatomy content delivered using the metaverse?*

5 = Very well understood

4 = Well understood

3 = Neutral

2 = Poorly understood

1 = Not understood at all.

2. *How did you feel about the class format in the metaverse environment?*

5 = Very positive

4 = Positive

3 = Neutral

2 = Negative

1 = Very negative.

3. *Would you like to participate in a metaverse-based anatomy session again in the future?*

5 = Strongly yes

4 = Yes

3 = Neutral

2 = No

1 = Strongly no.

4. *Compared with a traditional classroom lecture, what did you perceive as advantages of the metaverse-based class?*
5. *What did you perceive as disadvantages of the metaverse-based class?*
6. *Please provide specific impressions and/or suggestions for improvement regarding today’s session.*

Note: The original questionnaire was administered in Japanese.
