# Supplementary material for: Metaverse‐based virtual reality and spatial learning in cervicofacial anatomy: A curriculum‐embedded comparative study in undergraduate medical students
Source: Anat Sci Educ. 2026 Jul 6;19(9):1452–9. doi: 10.1002/ase.70277 (PMC13545329; doi:10.1002/ase.70277)
Supplement: Supplementary file 3 [file ASE-19-1452-s004.docx]

# Supplemental Data 1. Anatomy Comprehension Test Items

Instructions:
Please select the single best answer for each question. Mark your choice clearly.

1. Relative Position of the Cochlea

Where is the cochlea located in relation to the semicircular canals?

- Anterosuperior
- Posterosuperior
- Anteroinferior
- Posteroinferior

2. Facial Nerve and Semicircular Canal

Which semicircular canal is closest to the facial nerve?

- Superior semicircular canal
- Posterior semicircular canal
- Anterior semicircular canal
- Lateral semicircular canal

3. Course of the Chorda Tympani Nerve

Where does the chorda tympani nerve pass in relation to the ossicles?

- Between the tympanic membrane and the malleus
- Between the malleus and the incus
- Between the incus and the stapes
- Between the stapes and the inner ear

4. Connection of the Stapes Footplate

The footplate of the stapes communicates with which structure of the inner ear?

- Anterior semicircular canal
- Posterior semicircular canal
- Scala tympani of the cochlea
- Scala vestibuli of the cochlea

5. Location of the Carotid Canal in the Tympanic Cavity

Where is the carotid canal located within the tympanic cavity?

- Anterior to the hypotympanum
- Posterior to the hypotympanum
- Anterior to the epitympanum
- Posterior to the epitympanum

6. Most Anterior Paranasal Sinus

Which paranasal sinus is located most anteriorly?

- Frontal sinus
- Maxillary sinus
- Ethmoidal sinus
- Sphenoidal sinus

7. Structure in the Posterior Superior Nasal Septum

Which structure is located in the posterior superior portion of the nasal septum?

- Anterior nasal aperture
- Choana
- Nasopharynx
- Nasal cavity

8. Structure Located Posterior to the Orbit

Which structure is located posterior to the orbit?

- Frontal sinus
- Ethmoidal sinus
- Sphenoidal sinus
- Maxillary sinus

9. Structure Running on the Deep Surface of the Sternocleidomastoid Muscle

Which structure runs along the deep surface of the sternocleidomastoid muscle?

- Carotid artery
- Internal jugular vein
- Subclavian artery
- External jugular vein

10. Level of Bifurcation of the Common Carotid Artery

At approximately what vertebral level does the common carotid artery bifurcate into the internal and external carotid arteries?

- C1–C2 vertebral level
- C3–C4 vertebral level
- At the level of the cricoid cartilage
- At the level of the thyroid gland

11. Relationship Between the Subclavian Artery and Vein

What is the relationship between the subclavian artery and the subclavian vein?

- Artery anterior, vein posterior
- Artery inferior, vein superior
- Artery posterior, vein anterior
- None of the above

12. Approximate Level of the Thyroid Gland

Which of the following best describes the vertical extent of the thyroid gland?

- From the hyoid bone to the thyroid cartilage
- From the thyroid cartilage to the cricoid cartilage
- From the cricoid cartilage to the sternum
- From C3 to C5 vertebral level
